# Study of the road traffic noise in Erzurum-Turkey

**DOI:** 10.1186/1735-2746-9-22

**Published:** 2012-12-11

**Authors:** Mahir Gökdag

**Affiliations:** 1 Department of Civil Engineering, Faculty of Engineering, Ataturk University, Erzurum, Turkey

**Keywords:** Traffic noise, Noise pollution, Vehicle noise, Sound level, Environment, Noise

## Abstract

Road traffic noise affects a large number of people, especially in urban areas and is generally a major source of complaints. This paper refers to a study of the problem of traffic noise on roads which have been transformed into streets in the city of Erzurum. Noise levels are measured and the impacts suffered by the community are documented. Manual noise measurements were made along 12 streets exploring sources of maximum noise levels. Noise from different types of vehicles driven in a realistic way in inner city traffic was measured. The results from the measurements showed that, of 750 measured events, approximately 5% of the vehicles exceeded 70 dB(A) and less than 2% exceeded 80 dB(A) maximum noise level. In summary, the result showed that the most important vehicle component as regards the maximum noise level in inner city traffic was a medium-weight vehicle. Among the higher noise levels measured (>80 dB(A)) this type of vehicle was dominant.

## Introduction

In modern times, noise is recognized as a serious health problem. Road traffic is the most significant source in the city and is one of the most pervasive types of noise pollution and generally considered more intrusive than other types of noise such as industrial noise, airport and community noise. The main urban transport problems include street injuries and fatalities, congestion, inadequate public transport and pollution. Air and noise pollution are the two undesirable impacts of the vehicular traffic and are especially important in urban areas [1].

Noise pollution caused by traffic has become a big social problem and will become more serious and widespread. Over a number of years, investigations in different countries have shown that noise affects different activities and causes sleep disturbance and poorer life quality [2,3]. Noise pollution has a detrimental effect upon the learning and attainments of school children. Poor acoustical conditions and high noise levels can cause many problems for the instructors and students. The acoustical indices and conditions of classrooms are important factors in the learning achievement of students [4].

Noise is unacceptable level of sound that creates annoyance, hampers mental and physical peace, and may induce severe damage to the health. Along with the increasing degree of air and water pollution, noise pollution is also emerging as a new threat to the inhabitants of Sylhet City [5]. Noise effects include various impacts on mental and physical health and disturbance of daily activities, which may, lead to perception of annoyance, causing hearing loss, cardiovascular problems as well as affecting task performance [6].

Investigations have studied the extent of annoyance among persons exposed to different types and levels of environmental noise [7]. Some studies have suggested that the noise level and number of vehicles to be treated as independent variables instead of counting them into a single index. Results of these investigations have shown that the optimal way to express noise exposure is to describe it as the number of events over a certain noise level (70 dB(A)) and the maximum noise level that occurs three to five times per a 13 hour period. The number of events is important for the extent of annoyance, but only up to a certain point after which a further increase in the number of events does not lead to a further increase in the extent of annoyance [8-10]. Stosic *et al.,* investigated the effects of noise on sleep in an urban population and the relation between noise sensitivity and sleep disturbance [11]. Jakovljevic *et al.* were to determined principal factors for the onset of noise annoyance in an urban population and to assessed their predictive value [12].

The goal of this survey was to determine the source of maximum noise levels in city traffic and involved manual measurements of the highest noise levels from 12 different points. The study was conducted in the city of Erzurum (400,000 inhabitants), located in Eastern Turkey. Measurements were also made of the noise levels of different types of vehicles, driven under realistic and controlled conditions at different speeds.

## Materials and methods

### Road traffic noise measurements

Extensive road traffic noise measurements have been carried out recently by the authors at 12 sites covering different types of roads with different width, number of vehicles/hour, and speed. The measurements were performed by one person for a period of one hour, during non-rush hours, at some time between 7.30 and 20.30, using a microphone on a stand at a level of 1.5 m above the ground. The maximum level was read manually on a Brüel and Kjaer noise level analyzer (B and K 4426) in the position dB(A) FAST [13]. The distance to the nearest driving lane was 2 m. The measurement person simultaneously classified the type of vehicle under one of the following classes: private car, delivery van, medium-weight vehicle, heavy-weight vehicle and bus. For some calculations, the vehicles were divided into light vehicles (private car, delivery van) and heavy vehicles (medium-weight vehicle, heavy-weight vehicle and bus). Then, the passenger car equivalent for the traffic density was estimated by the divided vehicles [14].

The vehicles were driven past Brüel and Kjaer model placed in a free field at a height of 1.5 meters and a distance of 4 and 6 meters from the right side of the vehicles. The maximum noise level in dB(A) with the time constant FAST was measured with a noise level analyzer Brüel and Kjaer model when driving at different speeds past the measuring point. When evaluating the results, particular emphasis was placed on the maximum noise level of 80 dB(A). This level has not been previously suggested as a guideline for road traffic noise.

### Definitions

In this section some terms used in this survey are shortly explained:

*Sound pressure:* It is the pressure that reaches the tympana of human and animal ears, caused by the oscillating movement of the medium molecules. It is given in Pascal (Pa).

*Sound pressure level*: The reference sound pressure is the minimum sound pressure that can be perceived by the human ear. Its value is equivalent to 20 μPa (20×10^-6^ Pa).

(1) $$\mathrm{SPL}=10\log\left(\frac{\mathrm{pre}s^{2}}{\mathrm{refpre}s^{2}}\right)$$

Where *pres*=sound pressure; *refpres* = reference sound pressure. The unit used is the decibel (dB), submultiples of the bel (B).

*dB(A):* The ear’s sensitivity to sound is dependent on the frequency of the sound being detected. Frequencies at the extreme of the hearing frequency range are not detected as well as frequencies in the middle of the range. To account for this when measuring sound, a weighting curve is used to place more emphasis on frequencies to which humans are more susceptible. The “A” weighting curve is generally used for the purpose of measuring sound levels. The sound weighted by 25 the “A” curve approaches the perception of the human ear and its value is given by dB(A). Some typical values: 35 dB(A)-libraries; 65 dB(A) - business offices with normal conversation; 75 dB(A) —street with intense traffic measured at the curb; 85 dB(A)-street with heavy lorries passing 6 m away [15]. Since the vehicle counts were assumed in three categories (cars, buses, and trucks) and their maximum legal noise levels are given as 75 dB(A) for cars, 85 dB(A) for buses and 85 dB(A) for trucks [16].

*Equivalent level (Leq):* The noise levels are variable over time, going up and down continuously, making it difficult to evaluate. In order to make things easier the equivalent level was defined as a continuous sound level that would produce the same effect on the human ear if compared to the actual noise observed during the measurement, with all the variations. So, the *L*eq can substitute by a single value all the variations of the noise level. The equivalent sound level is also given by dB (A).

## Results

The study was based on relatively few measurements, and the results cannot be interpreted as representative for city traffic in general. The results from the measurements show that, of 750 measured events, approximately 5% of the vehicles exceeded 70 dB (A) and less than 2% exceeded 80 dB (A) maximum noise level. Table 1 reports the proportion of different vehicles that emitted a maximum noise level below and above 80dB (A). The data shows that medium-weight vehicle had the highest proportion of vehicles emitting more than 80 dB(A).

**Table 1:** Noise levels from: passenger cars (C), vans (V), medium-weight vehicle (MWV), buses (B), and heavy-weight vehicle (HWV)

**dB(A)**	**C**		**V**		**MWV**		**B**		**HWV**	
	**N**	**(%)**	**n**	**(%)**	**N**	**(%)**	**n**	**(%)**	**n**	**(%)**
<80	110	93	30	96	26	53	20	86	11	91
>80	9	7	3	4	21	47	5	14	5	9

**Figure 1:**
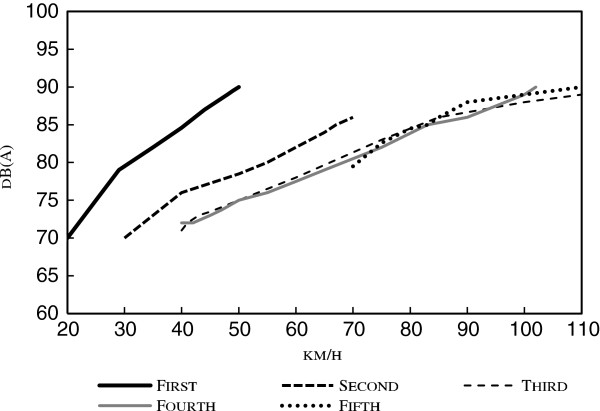
Noise level from car in relation to gear and speed.

The results of measurements made of a car driven at high speed in different gears are presented in Figure 1. The figure shows that driving in low gear at a high speed caused a high noise level. The noise increase was less marked when the car was driven at high speed in a higher gear owing to the dominant noise from the tires and the road.

**Figure 2:**
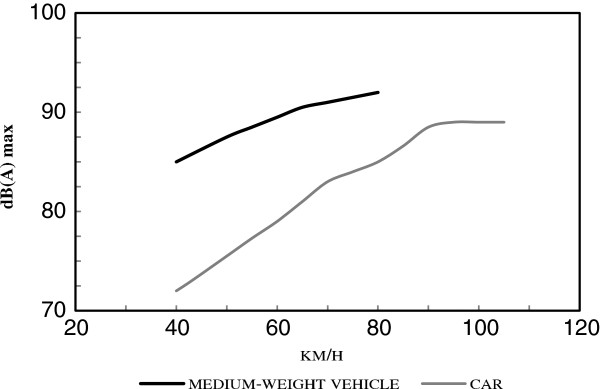
Noise level emitted by a car and medium-weight vehicles at normal speed in different gears at distance of 2M.

Figure 2 presents a comparison of noise levels from a car and a medium-weight vehicle driven at normal speed. It can be seen that the medium-weight vehicle was about 10 dB noisier at speeds of 35–45 km/hour. Disturbance caused by noise is one of the most important environmental health consequences of the transport apparatus.

## Discussion

The paper reviewed the various effects of road traffic noise on people's well-being with special emphasis on annoyance. According to the World Health Organization [17], *“Health is a state of complete physical, mental and social well-being”* Noise is, in this respect, more than just a nuisance, and it constitutes a danger that is real to people's health by producing both physical and psychological stress [9,12]. On the other hand, high levels of annoyance in individuals between 30 and 49 years of age, in comparison with those younger than 30 or older than 49, could be due to personality traits rooting in higher work and family responsibilities [18].

The study was based on relatively few measurements, and the results cannot be interpreted as representative for city traffic in general. Measures can be taken against the noisiest vehicle types specifically, and the noise load can be limited by introducing noise bans for particular streets in which vehicles that emit greater than a certain noise level, would not be allowed use of the street. The results show that relatively few vehicles emitted high maximum noise levels. It has previously been suggested that maximum 80 dB (A) should be the guideline value for environmental noise-induced annoyance. This suggests that important improvements in city traffic noise situation could be achieved by focusing attention on a relatively low number of vehicles.

The origin of the maximum noise levels was mainly medium-weight vehicles and only seldom passenger cars or heavy vehicles. This may be due to an aggressive driving behavior in low gears or insufficient noise reduction; age and maintenance may also play a role.

If the results of the present study are generally applicable, improvements in the noise situation should thus be directed mainly towards these vehicles and should be emphasized on better driving disciplines, improved noise control and/or maintenance.

In summary, the results show that medium-weight vehicles are an important source of high maximum noise levels in city traffic. The results further suggest that it is feasible to remedy the environmental discomfort caused by city traffic with rather limited actions towards particularly noisy vehicles. Human bodies react automatically and unconsciously to loud noises. In a world where people are constantly exposed to noise, the cumulative effects of noise on people may be quite extensive.

## Competing interests

The author declare that he had no competing interests.
